# Le délai diagnostique des cancers pédiatriques et le niveau socio-économique du ménage au Burkina Faso: une étude transversale descriptive et exploratoire

**DOI:** 10.11604/pamj.2026.53.44.46034

**Published:** 2026-01-29

**Authors:** Akiko Ida, Fla Kouéta, Sonia Douamba Kaboret, Isso Ouédraogo, Olivia Marie Ouedraogo, Roger Zerbo, Pori Aïda Traoré

**Affiliations:** 1 École Supérieure des Sciences Humaines et Sociales, Université de Shimané, Matsue, Japon,; 2 JICA Ogata Sadako Research Institute for Peace and Development, Tokyo, Japan,; 3 Département de Pédiatrie, Centre Hospitalier Universitaire Yalgado Ouédraogo, Ouagadougou, Burkina Faso,; 4 Unité d'Oncologie Pédiatrique, Centre Hospitalier Universitaire Pédiatrique Charles de Gaulle, Ouagadougou, Burkina Faso,; 5 Service de Chirurgie, Centre Hospitalier Universitaire Pédiatrique Charles de Gaulle, Ouagadougou, Burkina Faso,; 6 Direction de la Prévention et du Contrôle des Maladies Non Transmissibles, Ministère de la Santé et de l'Hygiène Publique, Ouagadougou, Burkina Faso,; 7 Centre National de la Recherche Scientifique et Technologique, Ouagadougou, Burkina Faso,; 8 Unité de Formation et de Recherche en Sciences Humaines, Université Joseph Ki-Zerbo, Ouagadougou, Burkina Faso

**Keywords:** Cancer pédiatrique, délai diagnostique, enfant, niveau socio-économique, oncologie, inégalités sociales de santé, système de santé, médecine traditionnelle, Burkina Faso, Afrique subsaharienne, Childhood cancer, diagnostic delay, children, socio-economic status, oncology, health inequalities, health systems, traditional medicine, Burkina Faso, sub-Saharan Africa

## Abstract

Le diagnostic tardif constitue une cause principale de la mortalité élevée des cancers pédiatriques dans les pays à revenu faible ou intermédiaire. Cette étude vise à mesurer les délais diagnostiques des cancers chez les enfants et à en examiner les relations avec le niveau socio-économique du ménage au Burkina Faso. Une étude transversale descriptive et exploratoire a été menée auprès de 36 enfants hospitalisés entre décembre 2023 et janvier 2024 dans les unités d'oncologie pédiatrique du CHU-YO et du CHUP-CDG à Ouagadougou. Les délais ont été calculés à partir des registres, des dossiers médicaux et des entretiens avec des accompagnants de l'enfant. Les corrélations ont été évaluées à l'aide du test de corrélation des rangs de Spearman ainsi que des tests de Mann-Whitney et de Kruskal-Wallis (p < 0,05). Le délai médian total de diagnostic était de 17,2 semaines [IQR: 22,9]. Le délai référentiel représentait la phase la plus longue (5,9 semaines). Les corrélations les plus fortes concernaient le délai pré-hospitalier, le niveau d'instruction des parents, l'accès à l'électricité, le statut de déplacé, la maîtrise du français et le nombre de pièces du logement (ρ = −0,37 à −0,47; p < 0,05). Près de la moitié des enfants (47,2%) avaient consulté la médecine traditionnelle. Les délais diagnostiques des cancers pédiatriques au Burkina Faso demeurent substantiels et sont liés aux contraintes du système de santé et aux inégalités socio-économiques. Le renforcement des capacités des structures de premier recours, un appui social aux ménages vulnérables et une meilleure compréhension du recours à la médecine traditionnelle seront nécessaires.

## Abstract (en)

Late diagnosis is a major cause of high mortality from pediatric cancers in low- and middle-income countries. This study aims to measure diagnostic delays in childhood cancers and to examine their relationship with household socio-economic status in Burkina Faso. We conducted a descriptive and exploratory cross-sectional study among 36 hospitalized children between December 2023 and January 2024 in the pediatric oncology units of CHU-YO and CHUP-CDG in Ouagadougou. Diagnostic delays were calculated using registries, medical records, and interviews with children's caregivers. Correlations were assessed using Spearman, Mann-Whitney and Kruskal-Wallis tests (p < 0.05). The median total diagnostic delay was 17.2 weeks [IQR: 22.9]. The referral interval represented the longest phase (5.9 weeks). The strongest correlations concerned the pre-hospital interval and parental educational level, access to electricity, displacement status, proficiency in French, and number of rooms in the home (ρ = −0.37 to −0.47; p < 0.05). Nearly half of the children (47.2%) had consulted traditional healers. Diagnostic delays for pediatric cancers in Burkina Faso remain substantial and are linked to health-system constraints and socio-economic inequalities. Strengthening the capacity of primary care facilities, providing social support to vulnerable households and improving understanding of the use of traditional medicine will be necessary.

## Introduction

Le diagnostic tardif constitue l'une des principales causes de la mortalité élevée chez les enfants atteints de cancer dans les pays à revenu faible ou intermédiaire (PRFI) [1]. Le délai diagnostique et ses déterminants demeurent toutefois mal connus dans les pays du Sahel, notamment au Burkina Faso, où les registres du cancer sont souvent incomplets. Le Burkina Faso, classé 186^e^ sur 193 pays selon l'indice de développement humain [2], dispose de deux unités principales d'oncologie pédiatrique (UOP): le Centre Hospitalier Universitaire Yalgado Ouédraogo (CHU-YO) et le Centre Hospitalier Universitaire Pédiatrique Charles de Gaulle (CHUP-CDG) à Ouagadougou. En 2019, 227 patients de moins de 15 ans y ont été pris en charge, soit 20% des cas attendus [3]. À titre d'exemple, le lymphome de Burkitt, l'un des cancers pédiatriques les plus fréquents dans la région, est souvent diagnostiqué à un stade avancé: en Afrique francophone, 77,5% des cas étaient au stade III ou plus entre 2016 et 2018 [4]. Cette étude visait à mesurer les délais diagnostiques des cancers pédiatriques et à en examiner les relations avec le niveau socio-économique (NSE) du ménage au Burkina Faso.

## Méthodes

**Conception de l'étude:** une étude transversale descriptive et exploratoire a été menée auprès des enfants de moins de 15 ans hospitalisés dans les UOP du CHU-YO et du CHUP-CDG entre décembre 2023 et janvier 2024.

**Participants et taille de l'échantillon:** les données ont été collectées à partir des registres hospitaliers, des dossiers médicaux et d'entretiens semi-directifs réalisés auprès des membres de la famille accompagnant l'enfant. Douze familles ont décliné la participation, trois enfants n'avaient pas eu de diagnostic confirmé et un adolescent avait plus de 15 ans, entraînant leur exclusion de l'enquête. Au total, trente-six enfants ont été inclus dans l'étude.

**Considérations éthiques:** l'étude a reçu l'approbation du Comité d'éthique pour la recherche en santé au Burkina Faso (n° 2023-10-238) et de l'Université de Shimané (n° 2024-201/MS/MESRI/CERS). Le consentement libre et éclairé a été obtenu de tous les participants.

**Variables:** les délais diagnostiques ont été définis comme suit: i) *délai pré-hospitalier:* entre l'apparition des premiers signes et la première consultation; ii) *délai référentiel:* entre la première consultation et l'arrivée à l'UOP; iii) *délai hospitalier:* entre l'entrée à l'UOP et la confirmation du diagnostic; iv) *délai diagnostique total:* somme des trois précédents. L'entrée à l'UOP a été utilisée comme point de départ hospitalier en raison de la régularité des données disponibles.

Le NSE du ménage a été évalué selon huit indicateurs: niveau d'éducation et profession des parents, accès à l'électricité et à l'eau, statut de déplacé interne, maîtrise du français, nombre de pièces du logement et moyen de transport. Les variables quantitatives ont été décrites par leur médiane et leur intervalle interquartile (IQR).

**Sources et mesures des données:** chaque variable a été codée de manière ordinale ou binaire, puis un score composite standardisé a été calculé et classé en tertiles (faible, moyen, élevé).

**Biais:** compte tenu du recours partiel aux entretiens rétrospectifs auprès des familles, un biais de mémoire ne peut être exclu.

**Méthodes statistiques:** les analyses ont été réalisées sous R (version 4.x) au moyen des tests de corrélation des rangs de Spearman et des tests de Mann-Whitney et de Kruskal-Wallis, avec un seuil de significativité fixé à p < 0,05.

## Résultats

**Participants:** trente-six enfants ont été inclus dans l'étude.

**Données descriptives:** leurs caractéristiques socio-démographiques et cliniques sont présentées dans le Tableau 1. L'âge médian était de 4 ans [IQR: 3-7], et 52,8% étaient de sexe masculin. La majorité provenait de la région Centre (35%), et certains étaient venus de la Côte d'Ivoire (5%) et du Ghana (2,5%). Les parents étaient majoritairement sans instruction (pères 47,2%, mères 50,0%). L'absence d'électricité concernait 58,3% des ménages et celle d'eau potable 55,6%. Six familles (16,7%) étaient déplacées internes et 66,7% déclaraient parler français. La médiane du nombre de pièces de leur habitation était de 2 [IQR: 1-2] et la moto constituait le principal moyen de transport (91,7%).

**Tableau 1:** caractéristiques socio-démographiques et cliniques des enfants atteints de cancer hospitalisés au CHU-YO et au CHUP-CDG, Ouagadougou, Burkina Faso (n = 36)*

Variable	Résultats
**Caractéristiques de l’enfant**	
Sexe masculin	19 (52,8%)
Âge (années), médiane [IQR]	4 [3–7]
Type de cancer	Leucémie 8 (22,2 %); Lymphome de Burkitt 7 (19,4 %); Lymphome de Hodgkin 1 (2,8 %); Hépatoblastome 1 (2,8 %); Néphroblastome 11 (30,6 %); Rétinoblastome 7 (19,4 %); Rhabdomyosarcome 1 (2,8 %)
Groupe de cancer	Hémopathie maligne 16 (44,4%); Tumeur solide 20 (55,6%)
Recours à la médecine traditionnelle	Oui 17 (47,2%); Non 19 (52,8%)
**Caractéristiques du ménage**	
Niveau d'éducation du père	Aucun 17 (47,2%); Primaire 4 (11,1%); Secondaire 13 (36,1%); Supérieur 2 (5,6%)
Niveau d'éducation de la mère	Aucun 18 (50,0%); Primaire 8 (22,2%); Secondaire 8 (22,2%); Supérieur 2 (5,6%)
Profession du père	Informel 25 (69,4%); Salarié 7 (19,4%); Professionnel 1 (2,8%); Sans emploi 3 (8,3%)
Profession de la mère	Informelle 21 (58,3%); Salariée 6 (16,7%); Professionnelle 1 (2,8%); Sans emploi 8 (22,2%)
Eau potable disponible	Oui 16 (44,4%); Non 20 (55,6%)
Électricité disponible	Oui 15 (41,7%); Non 21 (58,3%)
Nombre de pièces du logement, médiane [IQR]	2 [1–2]
Moyen de transport principal	Moto 33 (91,7%); Vélo 2 (5,6%); À pied 1 (2,8%)
Statut de déplacé interne	Oui 6 (16,7%); Non 30 (83,3%)
Parle le français	Oui 24 (66,7%); Non 12 (33,3%)
*Période: décembre 2023 – janvier 2024. IQR : intervalle interquartile. Les pourcentages peuvent ne pas totaliser 100% en raison d’arrondis.	

Les cancers les plus fréquents dans l'étude étaient le néphroblastome (30,6%), la leucémie (22,2 %), le lymphome de Burkitt (19,4 %) et le rétinoblastome (19,4 %).

**Données de résultats:** le délai diagnostique médian total était de 17,2 semaines [IQR: 22,9] (Tableau 2). Le délai référentiel représentait la phase la plus longue (5,9 semaines [IQR: 15,7]), suivi du délai hospitalier (1,1 [IQR: 1,9]) et du délai pré-hospitalier (0,6 [IQR: 7,3]). Près de la moitié des enfants (47,2%) avaient consulté la médecine traditionnelle avant l'hôpital, 47,2% avaient fréquenté plusieurs hôpitaux et 38,9% alternaient entre structures publiques et privées.

**Tableau 2:** délais diagnostiques en semaines chez les enfants atteints de cancer hospitalisés au CHU-YO et au CHUP-CDG, Ouagadougou, Burkina Faso (n = 36)*

Variable	Médiane	IQR
Délai pré-hospitalier	0,6	7,3
Délai référentiel	5,9	15,7
Délai hospitalier	1,1	1,9
Délai diagnostique total	17,2	22,9

* Période: décembre 2023 - janvier 2024. IQR: intervalle interquartile. Le délai pré-hospitalier correspond au délai entre l'apparition des premiers signes et la première consultation. Le délai référentiel correspond au délai entre la première consultation et l'arrivée à l'unité d'oncologie pédiatrique (UOP). Le délai hospitalier correspond au délai entre l'admission à l'UOP et la confirmation diagnostique. Le délai diagnostique total correspond à la somme de ces trois composantes.

**Principaux résultats:** le délai pré-hospitalier était significativement associé à plusieurs indicateurs du NSE (Tableau 3). Les enfants issus de familles moins instruites, ne parlant pas le français ou vivant dans des conditions précaires — statut de déplacement interne, absence d'électricité, nombre réduit de pièces du logement — présentaient des délais significativement plus longs avant la première consultation (ρ = −0,37 à −0,47, p < 0,05), traduisant des inégalités d'accès initial au système de soins.

**Tableau 3:** corrélations entre les indicateurs socioéconomiques du ménage et les délais diagnostiques chez les enfants atteints de cancer hospitalisés au CHU-YO et au CHUP-CDG, Ouagadougou, Burkina Faso (n = 36)*

Indicateur	Délai concerné	ρ (rho)	p	Interprétation courte
Éducation du père	Délai pré-hospitalier	−0.40	0.016	faible éducation → délai
Éducation de la mère	Délai pré-hospitalier	−0.37	0.024	idem
Électricité disponible	Délai pré-hospitalier	−0.42	0.010	précarité matérielle → délai
Nombre de pièces du logement	Délai pré-hospitalier	−0.45	0.006	précarité matérielle → délai
Statut déplacé (non-déplacé = 1)	Délai pré-hospitalier	−0.39	0.020	vulnérabilité sociale et géographique → délai
Parle le français	Délai pré-hospitalier	−0.47	0.004	barrière linguistique forte → délai

* Période: décembre 2023 – janvier 2024. Corrélation des rangs de Spearman (ρ). Seuil de significativité fixé à p < 0,05.

**Autres analyses:** aucune différence significative n'a été observée entre les tertiles du score composite (p = 0,139), ce qui suggère que les indicateurs individuels sont plus discriminants que le score socio-économique composite dans cet échantillon de petite taille.

## Discussion

Notre étude met en évidence un délai diagnostique médian de 17,2 semaines, comparable à celui rapporté dans la littérature en Afrique subsaharienne (6-16 semaines) [5-9] bien qu'il demeure plus long. La phase référentielle est la plus longue, ce qui suggère des difficultés d'orientation vers les UOP et la complexité de reconnaître des signes précoces d'un cancer au niveau des structures de premier recours. Ces comparaisons doivent toutefois être interprétées avec prudence, notre définition du délai référentiel incluant l'arrivée à l'UOP, ce qui peut allonger cette composante par rapport aux définitions habituellement utilisées.

Les IQR étendus reflètent une forte hétérogénéité des trajectoires diagnostiques, liées à la disponibilité des examens, aux charges de travail des services ou à la complexité clinique des cas.

Les déterminants socio-économiques influencent principalement la phase pré-hospitalière: les enfants issus de familles moins instruites, en situation de déplacement interne, vivant dans des conditions sociales et matérielles précaires ou ne maîtrisant pas le français présentaient des délais plus longs avant la première consultation. Ces inégalités d'accès initial sont renforcées par le recours fréquent à la médecine traditionnelle, souvent sous-déclaré, retardant l'accès au système de santé. Ces constats rejoignent ceux de Cotache-Condor *et al*. [10], indiquant que les déterminants sociaux et l'utilisation de la médecine traditionnelle peuvent contribuer au retard du diagnostic. Pour améliorer les délais diagnostiques, il faudra donc adopter une approche intégrée qui porte à la fois sur le soutien aux établissements de soins primaires et sur l'accompagnement des ménages vulnérables.

Bien qu'un score composite du NSE ait été construit, sa classification en tertiles s'est révélée instable en raison de la petite taille de l'échantillon; les indicateurs individuels se sont avérés plus robustes pour explorer les inégalités sociales.

**Limites:** cette étude présente plusieurs limites. Taille réduite de l'échantillon (n = 36) limitant la généralisation; inclusion uniquement des enfants hospitalisés, excluant les cas ambulatoires; qualité variable des données dans les registres hospitaliers; dates fournies par les accompagnants, exposant à un risque de biais déclaratif; probable sous-déclaration du recours à la médecine traditionnelle; période de collecte durant l'hivernage, marquée par des contraintes économiques pour les ménages agricoles, pouvant réduire la fréquentation hospitalière et l'absence de prise en compte des distances entre la résidence et l'UOP et contraintes de transport.

## Conclusion

Cette étude montre que les délais diagnostiques des cancers pédiatriques au Burkina Faso demeurent substantiels, le délai référentiel constituant la phase la plus prolongée du parcours de soins. Les résultats indiquent que les obstacles à un diagnostic rapide relèvent à la fois de contraintes organisationnelles au sein du système de santé et de déterminants socio-économiques influençant le recours initial aux soins. La réduction de ces délais suppose des stratégies intégrées, combinant le renforcement des capacités des structures de premier recours, un appui social aux ménages vulnérables et une meilleure compréhension du recours à la médecine traditionnelle. Des recherches futures, incluant des dimensions géographiques et qualitatives, permettront d'affiner ces observations et d'adapter les interventions aux réalités locales.

### Etat des connaissances sur le sujet

- Le diagnostic tardif constitue une cause majeure de la mortalité élevée des cancers pédiatriques dans les pays à revenu faible ou intermédiaire;
- Au Burkina Faso, les délais diagnostiques et leurs relations avec le niveau socio-économique du ménage restent peu documentés.

### Contribution de notre étude à la connaissance

- Cette étude fournit l'une des rares estimations détaillées du délai diagnostique des cancers pédiatriques au Burkina Faso;
- Elle met en évidence la double influence des inégalités socio-économiques, agissant avant l'accès au système de santé, et des contraintes structurelles, ralentissant l'orientation vers les unités spécialisées;
- Elle souligne la nécessité d'aborder les délais diagnostiques dans une perspective multidimensionnelle, intégrant les déterminants sociaux, économiques, linguistiques, culturels et institutionnels, afin d'identifier les leviers d'un diagnostic plus précoce.

## Remerciements

Les auteurs expriment leur gratitude au Ministère de la Santé et de l'Hygiène publique du Burkina Faso, au CHU Yalgado Ouédraogo et au CHUP Charles de Gaulle pour avoir autorisé et soutenu cette recherche. Nous tenons également à remercier le Dr Gabrielle Chantal Bouda et le Dr Rolande Kaboré pour leur aide précieuse dans l'accès au registre hospitalier. En particulier, nous souhaitons exprimer notre reconnaissance à M. Ikuo Takizawa pour ses relectures et ses commentaires constructifs, ainsi qu'au Dr Satoko Suzuki pour son appui administratif. Enfin, nous adressons nos sincères remerciements à toutes les personnes qui ont accepté de participer à cette étude - notamment les accompagnants, les professionnels de santé, les travailleurs sociaux et l'association Enfants sans cancer du Burkina Faso - de même qu'à l'évaluateur anonyme et à l'équipe éditoriale du journal.
